# Characterization of Microbiota in Children with Chronic Functional Constipation

**DOI:** 10.1371/journal.pone.0164731

**Published:** 2016-10-19

**Authors:** Tim G. J. de Meij, Evelien F. J. de Groot, Anat Eck, Andries E. Budding, C. M. Frank Kneepkens, Marc A. Benninga, Adriaan A. van Bodegraven, Paul H. M. Savelkoul

**Affiliations:** 1 Department of Pediatric Gastroenterology, VU University Medical Center, Amsterdam, The Netherlands; 2 Department of Gastroenterology and Hepatology, VU University Medical Center, Amsterdam, The Netherlands; 3 Department of Medical Microbiology & Infection Control, VU University Medical Center, Amsterdam, The Netherlands; 4 Department of Pediatric Gastroenterology, Emma Children’s Hospital, Academic Medical Center, Amsterdam, The Netherlands; 5 Department of Gastroenterology, Geriatrics, Internal and Intensive Care Medicine (Co-MIK), Zuyderland Medical Center, Heerlen-Sittard-Geleen, The Netherlands; 6 Department of Medical Microbiology, Maastricht University Medical Center, Maastricht, The Netherlands; University of Minnesota, UNITED STATES

## Abstract

**Objectives:**

Disruption of the intestinal microbiota is considered an etiological factor in pediatric functional constipation. Scientifically based selection of potential beneficial probiotic strains in functional constipation therapy is not feasible due to insufficient knowledge of microbiota composition in affected subjects. The aim of this study was to describe microbial composition and diversity in children with functional constipation, compared to healthy controls.

**Study Design:**

Fecal samples from 76 children diagnosed with functional constipation according to the Rome III criteria (median age 8.0 years; range 4.2–17.8) were analyzed by IS-pro, a PCR-based microbiota profiling method. Outcome was compared with intestinal microbiota profiles of 61 healthy children (median 8.6 years; range 4.1–17.9). Microbiota dissimilarity was depicted by principal coordinate analysis (PCoA), diversity was calculated by Shannon diversity index. To determine the most discriminative species, cross validated logistic ridge regression was performed.

**Results:**

Applying total microbiota profiles (all phyla together) or per phylum analysis, no disease-specific separation was observed by PCoA and by calculation of diversity indices. By ridge regression, however, functional constipation and controls could be discriminated with 82% accuracy. Most discriminative species were *Bacteroides fragilis*, *Bacteroides ovatus*, *Bifidobacterium longum*, *Parabacteroides* species (increased in functional constipation) and *Alistipes finegoldii* (decreased in functional constipation).

**Conclusions:**

None of the commonly used unsupervised statistical methods allowed for microbiota-based discrimination of children with functional constipation and controls. By ridge regression, however, both groups could be discriminated with 82% accuracy. Optimization of microbiota-based interventions in constipated children warrants further characterization of microbial signatures linked to clinical subgroups of functional constipation.

## Introduction

Chronic constipation is a common condition, affecting approximately 3% of children in the Western world [1]. In more than 90% of these children, no underlying organic cause can be found [2]. The diagnosis of functional constipation is based on the Rome-III diagnostic criteria [3]. The etiology of functional constipation is considered multifactorial and has not been fully clarified yet. Withholding behavior is considered one of the major causative mechanisms, next to psychological factors and social conditions [4,5]. In several studies, intestinal gut microbiota has been shown to influence gastrointestinal motility. Microbial disturbance has therefore been linked to the development of functional constipation and manipulation of the intestinal microbiota with prebiotics and probiotics has increasingly been considered a target for therapeutic interventions [6,7,8,9,10]. In several randomized controlled trials the efficacy of probiotics in functional constipation has been studied, using various probiotic mixtures and concentrations, with contradictory outcomes [11,12,13]. To assess which (combination of) probiotic strain(s), if any, might be beneficial in rationale-based therapeutic strategies for functional constipation, detailed delineation of gut microbiota composition is pivotal [14]. Surprisingly, knowledge regarding possible constipation-defining intestinal microbial signatures is scarce, especially in children [6].

Therefore, the aim of this study was to describe the composition and diversity of the intestinal microbiota in pediatric functional constipation in comparison with healthy controls, based on microbial profiling of the total gut microbiota with the PCR-based technique IS-pro [15,16].

## Methods

### Subjects

In this prospective study, performed between July 2012 and July 2014, eligible patients were children with refractory symptoms of constipation referred by general pediatricians from different hospitals in the Netherlands to the VU University Medical Center and the Academic Medical Center (both tertiary referral centers, located in Amsterdam, the Netherlands). Inclusion criteria were age between 4–18 years and diagnosis of functional constipation according to the Rome III criteria [3]. Exclusion criteria were culture-proven infectious colitis; use of antibiotics, corticosteroids or immunosuppressive therapy within three months prior to inclusion; a diagnosis of gastro-intestinal disease (such as celiac disease and inflammatory bowel disease) or neurological conditions (such as spina bifida and Hirschsprung’s disease) or anatomic abnormalities of the gastro-intestinal tract. Also children with Irritable Bowel Syndrome according to Rome III criteria were excluded. Controls fulfilled similar exclusion criteria as the study group. A formal power analysis could not be done, since no sufficient data on microbiota analysis using molecular detection techniques in constipated children were available. Totally, 76 children with functional constipation were included consecutively and at inclusion they were instructed to discontinue all prescribed laxatives for a period of four weeks prior to collection of the study sample, in order to limit the risk of a type I error. All study subjects and controls were asked to provide information on stool pattern and consistency, use of laxatives and other medication, and duration of symptoms of constipation. Children were provided a sterile plastic container and were instructed to collect and store a fecal sample in the domestic freezer directly following defecation (-20°C). After transport, samples were kept frozen at -20°C until further processing. In order to construct a matched control group optimally reflecting health state, fecal samples collected by 61 healthy Dutch children, who participated in a previous study on intestinal microbiota composition and dynamics, were used [16]. In that study on intestinal microbial dynamics, fecal samples were collected each week for six weeks and an additional follow-up sample was collected after eighteen months. All 61 baseline samples were selected to serve as controls in the present study. An identical protocol was used for collection, storage, transport, handling and microbiota analysis of these fecal samples. The present study was approved by the Ethics Committees of VU University Medical Center and the Academic Medical Center, and was performed in accordance with the ethical standards laid down in the 1964 Declaration of Helsinki and its subsequent amendments. Both ethical committees concluded that the Medical Research Involving Human Subjects Act (WMO) did not apply on this study. Informed consent (verbal) by the parents was documented.

### Sampling preparation, DNA extraction, and polymerase chain reaction

All fecal samples were prepared and analyzed by means of standard IS-pro procedure [15,16]. IS-pro is a DNA-based microbiota profiling technique, based on the identification of species-specific length polymorphisms of the interspacer (IS) region and phylum-specific sequence polymorphisms of 16S rDNA. In short, 300 μl lysis buffer was added to a Eppendorf container containing 0.5 gr frozen fecal sample. This mixture was vortexed and subsequently shaken for 5 minutes at room temperature. The containers were centrifuged for 2 minutes at 13,000g. The resulting supernatant was subsequently transferred to an empty DNA isolation vial, followed by adding magnetic silica beads according to the routine protocol. Bacterial DNA was isolated by a standard automated isolation procedure (EasyMag, Biomereux, Marcy l’Etoile, France). The resulting total DNA was eluted in 110 μl buffer and stored at 4°C until use for PCR amplification. ISpro technique: Isolated DNA (10μl/PCR)was amplified in two normal standardized multiplex PCR amplifications: (1) *Firmicutes*, *Actinobacteria*, *Fusobacteria*, *Verrucomicrobia* (FAFV), *Bacteroidetes* and (2) *Proteobacteria*. The forward FAFV, *Bacteriodetes* and *Proteobacteria* primers contain different fluorescent labels for phylum identification. Further information on the used primers has been published previously [15,16]. After amplification, 5 μl of PCR product was mixed with 20 μl formamide and 0,2 μl Mapmaker 1500 ROX labeled size marker (custom made by BioVentures, Murfreesboro, TN, USA). Subsequently, PCR products were separated based on their different lengths in an ABI Prism 3130XL Genetic Fragment Analyzer (Applied Biosystems Carlsbad, California, USA). Overall, three levels of information are obtained: color of peaks discriminates detected peaks into the phyla *FAFV*, *Bacteroidetes* and *Proteobacteria*, together covering the major phyla present in the human gut. Measured length of the 16S–23S rDNA IS region, displayed by number of nucleotides, is used to identify bacteria at species level based on a database consisting of more than 1500 species and their corresponding IS lengths. Peak height, measured in relative fluorescence units (RFU), corresponds to the quantity of PCR product, reflecting the relative abundance of present species.

### Data analysis

Data were analyzed with the standard IS-pro proprietary software suite. Basically, for profile correlation, a log2 transformation was performed on peak heights for improved consistency of estimated correlation coefficient and improved detection of variation in less prominent species [11]. A clustered heat map was made by generating a correlation matrix of all log2 transformed profile data followed by clustering with the unweighted pair group method with arithmetic mean (UPGMA). Within-sample microbial diversity was calculated as the Shannon diversity index based on the resulting profiles using the R 2.15.2 software package. Diversity was calculated both per phylum and for overall microbial composition (by pooling the phyla *FAFV*, *Bacteroidetes* and *Proteobacteria* together). A p-value of < 0.05 was considered statistically significant. Sample compositions were compared by calculating cosine distances for log2 transformed data per phylum and for the phyla *FAFV*, *Bacteroidetes* and *Proteobacteria* combined. Dissimilarity in microbiota composition was depicted as principal coordinate analysis (PCoA), based on cosine distance measures.

Cross validated adaptive group-regularized (logistic) ridge regression model was performed for the prediction of the clinical status of the samples (constipation or control) with a 10-fold cross validation. This classifier uses an ℓ 2-penalty for regularization and allows for the structural use of co-data in order to improve predictive performance. The juxtapositions of interest in the classification exercise are informed by the results of the GlobalTesting exercise. This supervised classification learning model is used in order to identify patterns in complex high-dimensional data and to discriminate between groups. PLS Discriminant Analysis (PLS-DA) was performed to provide a quantitative estimate of the discriminatory power of each descriptor (species, in our case), which we used to rank the species importance in the model. By doing so, most discriminative species between the two study groups could be assessed. Data visualizations were performed with the Spotfire software package (TIBCO, Palo Alto, CA, USA).

To investigate whether different clinical characteristics of subjects with functional constipation corresponded with discriminative microbial signatures, we repeated the analysis described above per subgroup; microbial profiles of constipated children with and without withholding behavior were compared. Similarly, to determine whether frequency of bowel movements was associated with differences in intestinal microbiota composition, IS profiles of affected children with ≤ 2 bowel movements per week were compared to those with more than 2 bowel movements per week.

## Results

### Study population

In total 76 consecutive children meeting the Rome III diagnostic criteria of functional constipation were enrolled in this study. On inclusion, 65 children (86%) were on laxative treatment. All children temporarily discontinued the constipation-related medication prior to collection of the study sample.

### Healthy controls

Relevant findings from the study separately describing the intestinal microbiota composition in the control group were that microbial stability in healthy children varied per phylum, at both short-term and long-term intervals. In the same study a shared, age-independent microbiota core was observed, consisting of a limited number of species, predominantly belonging to the phylum *Bacteroidetes*. None of the 61 healthy controls suffered from gastro-intestinal problems. Four children reported abdominal pain in the past three months prior to inclusion, however, none of these children fulfilled the Rome III criteria of irritable bowel syndrome [16]. Complete patient characteristics of both study groups are displayed in Table 1.

**Table 1:** Subject characteristics.

		Functional Constipation	Controls
Number of subjects		76	61
Age (y) (median, range)		8.0 (4.2–17.8)	8.6 (4.1–17.9)
Male (%)		50	46
Bowel movements per week (%)			
	<1	15	-
	1–2	28	-
	>2	57	100
≥ 1 episode of fecal incontinence per week (%)		61	0
Presence of large fecal mass in rectum or abdomen (%)		36	NA
retentive posturing (%)		57	0
History of large diameter stools that may obstruct the toilet (%)		43	0
History of painful or hard bowel movements (%)		71	0
Abdominal pain in last 3 months prior to inclusion (%)⁰		79	7
Duration of symptoms before inclusion (months)		45 (range 5–172)	NA
Laxatives on inclusion (n) *			
	Polyethylene glycol 4000	53 (range 4–80 grams/day)	0
	Polyethylene glycol-electrolyte solution	2	0
	Bisacodyl	6	0
	Lactulose	7	0
	Enema	12	0
	Prucalopride	1	0
	None	11	61
	Insufficiently documented	2	0
Antibiotic use in first year of life (n)		9	13
Co-medication (n)			
	Methylphenidate	2	0
	Salbutamol	1	0
	Aripripazol	1	0
	Melatonin	1	0
	Birth control pills	0	1
	Probiotics	0	3
	Multivitamins	10	19

⁰ None of the children fulfilled the Rome III criteria of Irritable Bowel Syndrome

* All laxatives were discontinued by all subjects, four weeks prior to collection of the fecal samples

### Microbiota analysis

**Fig 1:**
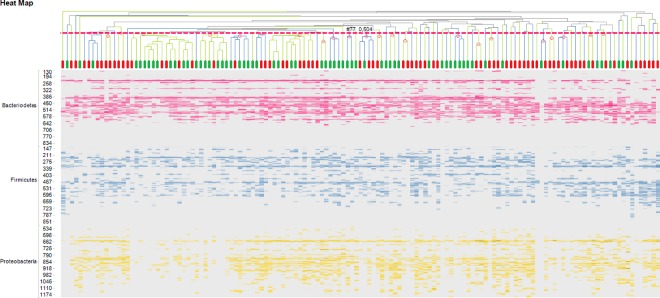
Clustered heat map with IS profiles of children with functional constipation and controls. Clustered heat map displaying IS profiles of 76 children with functional constipation and 61 healthy controls. Individual subjects are shown on the X axis; children with constipation in red, healthy controls in green. On the Y axis, IS fragment lengths are expressed (in number of nucleotides), corresponding with bacterial strain type (OTU). Blue peaks represent *Firmicutes*, *Actinobacteria*, *Fusobacteria*, *Verrucomicrobia (FAFV)*, red peaks represent *Bacteroidetes* and yellow peaks represent *Proteobacteria*. Intensity of colors reflect relative dominance of each indicated bacterial strain, grey signals represent less prevalent IS fragment lengths. No disease-specific clustering was observed, indicating that the groups could not be distinguished based on IS profiles using this unsupervised method. The most abundant OTUs in both study groups were observed within the phylum *Bacteriodetes*, corresponding to the species *Bacteroides vulgatis* (480 nt), *Alistipes putredinis* (220 nt) and *Alistipes finegoldii* (401 nt).

**Fig 2:**
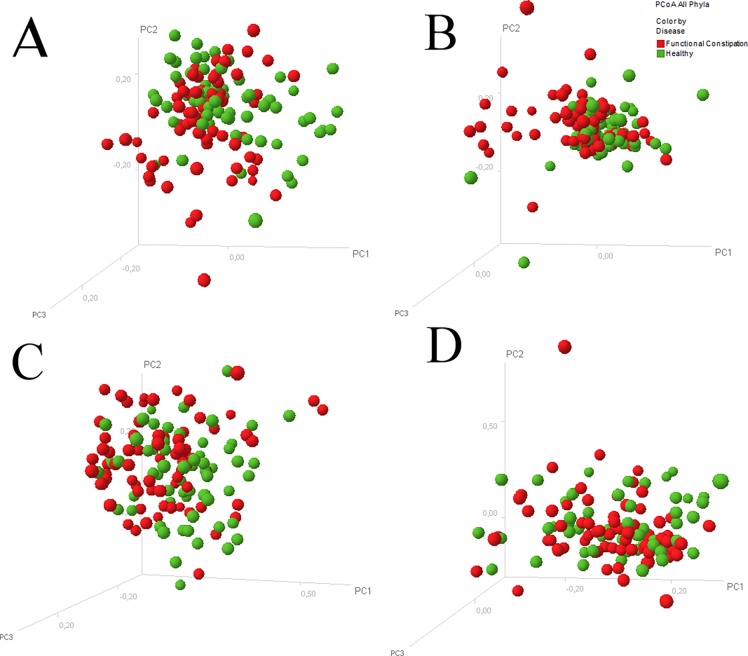
Principle coordinate analysis of microbial profiles of children with and without functional constipation. Principle coordinate analysis scatterplot displaying overall bacterial community composition, showing no separate clustering of microbial profiles of children with functional constipation (red dots) and controls (green dots) for all phyla together (A) and per phylum (B: B*acteroidetes*; C: *Firmicutes*, *Actinobacteria*, *Fusobacteria*, *Verrucomicrobia (FAFV)*; D: *Proteobacteria)*.

**Fig 3:**
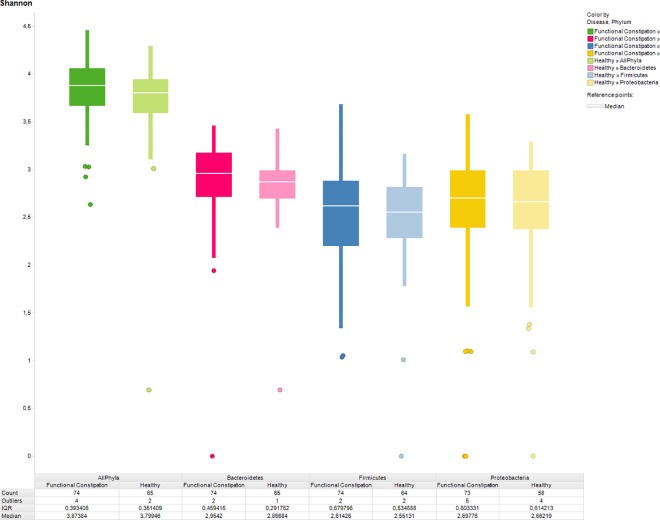
Diversity indices of children with functional constipation and healthy controls. Shannon diversity index of children with functional constipation (dark colors) and healthy controls (light colors), showing similar indices, both when taken all phyla together, as well as on phylum level. Green: all phyla taken together. Red: *Bacteroidetes*, blue: represent *Firmicutes*, *Actinobacteria*, *Fusobacteria*, *Verrucomicrobia (FAFV)*, yellow: *Proteobacteria*.

The most abundant species in the IS profiles of both study groups were observed within the phylum *Bacteriodetes*, presented by the species *Bacteroides vulgatus* (480 nt length position), *Alistipes putredinis* (220 nt) and *Alistipes finegoldii* (401 nt). A clustered heat map did not reveal disease-specific clustering, neither at phylum level nor at species level (Fig 1). PCoA revealed no segregation between children with functional constipation and healthy controls, neither for all phyla together nor per phylum (Fig 2). Likewise, Shannon diversity index was not statistically significantly different between the study group and the control group regarding diversity at the phylum level. Similar diversity indices were found for all phyla combined (functional constipation 3.9 (IQR 0.4); controls 3.8 (0.4); p = 0.13), as well as per phylum: *Bacteriodetes* (functional constipation 2.9; IQR 0.5; controls 2.9; IQR 0.3; p = 0.075); *FAFV* (functional constipation 2.6 IQR 0.7; controls 2.6 IQR 0.5; p = 0.75); *Proteobacteria* (functional constipation 2,7; IQR 0.6; controls 2.7; IQR 0.6; p = 0.62) (Fig 3).

**Fig 4:**
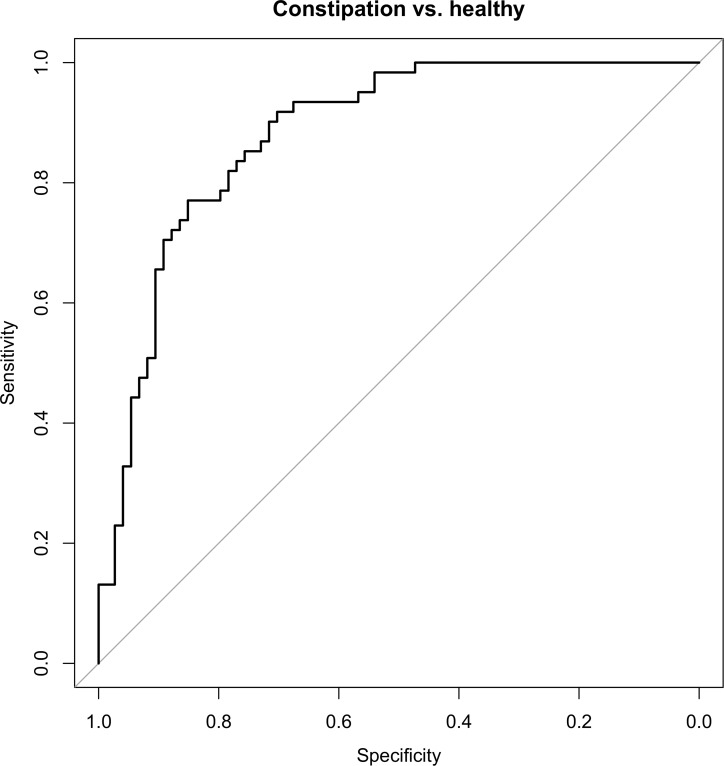
Receiver operating characteristic (ROC) curves for the discrimination of children with and without functional constipation. Receiver operating characteristic (ROC) curves summarizing the predictive power of the cross-validated logistic ridge regression model for clinical status per phylum and for all phyla combined.

By using the cross validated adaptive group-regularized (logistic) ridge regression model microbial profiles of children with constipation could be discriminated from matched controls (AUC ± 95% CI, sensitivity, specificity: 0.78 ± 0.08, 90%, 73%) with accuracy of 82% (Fig 4). Most discriminative species within the phyla *FAFV*, *Bacteriodetes* and *Proteobacteria* are shown in Table 2.

**Table 2:** Discriminative species between functional constipation and controls.

Bacteroidetes	FAFV	Proteobacteria
*Bacteroides fragilis* (528) ↑	*Bifidobacterium longum* (480) ↑	*Proteus mirabilis* (898) ↑
*Bacteroides ovatus* (600) ↑	*Ruminococcus spp*. (826) ↓	Unknown species (649) ↑
*Parabacteroides spp*. (507) ↑		
*Alistipes finegoldii* (401) ↓		

Discriminative species (nt) of the major phyla of the gut (*Firmicutes*, *Actinobacteria*, *Fusobacteria*, *Verrucomicrobia* (FAFV), *Bacteriodetes* and *proteobacteria*) between children with functional constipation and matched controls, as detected by ridge regression

↑ = increased abundance in functional constipation

↓ = decreased abundance in functional constipation; spp. = species

To assess whether clinical characteristics of constipated children were correlated with specific microbiota alterations, the analyses were repeated per subgroup. Profiles of constipated children with and without withholding behavior could not be discriminated. Furthermore, children with functional constipation with ≤ 2 bowel movement per week could not be differentiated from controls and from children with functional constipation with >2 bowel movements per week (data not shown).

Since 61% of children with functional constipation had fecal incontinence, a post hoc analysis was performed to compare IS profiles of children with and without fecal incontinence, showing that both subgroups could not be discriminated (data not shown).

## Discussion

In this prospective study, we have characterized the intestinal microbiota in a large cohort of children with functional constipation defined by Rome III criteria. Using a supervised statistical learning method affected children could be discriminated from healthy controls with 82% accuracy.

The interaction between intestinal microbiota and gastrointestinal transit has predominantly been studied in germ-free animals, demonstrating a clear association between absence of microbial colonization and disruption of gut motility [7,10,17,18,19]. While the exact pathophysiological mechanisms behind the interplay between gut microbes and motility still has to be elucidated, several possible mechanisms have been postulated. In a mouse model, it was demonstrated that microbes could modulate the expression of genes involved in motor apparatus responses [20]. Other explanations include pH dependent motility stimulation by fermentation products, osmotic effects of microbiota metabolites and intestinal distension by increased intraluminal gas production (carbon dioxide, hydrogen and methane) causing reflexive smooth muscle contractions [21]. Apart from these general mechanisms linking microbiota to gastrointestinal motility, several studies have aimed to link specific bacterial strains to (changes in) gut motility. *Bifidobacterium bifidum*, *Lactobacillus reuterii* and *Lactobacillus acidophilus* have been reported to promote gastrointestinal motility in animal studies, while *Escherichia* species may inhibit motility [22,23,24]. *In vitro* motility studies using human colon specimens, *Lactobacillus rhamnosus* GG and *Escherichia coli* Nissle 1917 have been reported to promote gastrointestinal motility and muscle cell contractility [21,25].

Slow transit constipation in children has indirectly been linked to microbial shifts, by detection of increased methane levels in exhaled breath, allegedly reflecting excessive intracolonic anaerobic archaeal activity, mainly attributed to *Methanobrevibacter smithii* [26,27]. In a recent study by Parthasarathy and colleagues, colonic mucosa-associated microbiota analysis could discriminate 25 adult patients with constipation from 25 healthy adults with 94% accuracy. Genera within the phylum *Bacteriodetes* were found to be more abundant in constipated subjects. Interestingly, only mucosal but not fecal microbiota emerged as predictive biomarker of constipation [28]. Due to these observations it has been assumed that probiotics might be beneficial in the treatment of functional constipation. However, in the majority of intervention studies using probiotics, gut microbiota composition was not assessed prior to and following administration of probiotics. Thus, any clinical effect on motility, supposedly ascribed to probiotic use, could therefore not be accredited to specific shifts in microbial composition. So far, only two small-scale studies have described the intestinal microbiome composition in children with functional constipation.

In a study on 28 affected children versus 14 controls using conventional culturing techniques, a statistically significant increase in colonic *Clostridium* and *Bifidobacterium* species was reported [6]. Similar to these findings, we observed an increased abundance of *Bifidobacteria* in constipated subjects, further classified on species level as *Bifidobacterium Longum*. Recently, fecal microbiota of eight constipated obese children has been compared with fourteen obese controls by 16S rRNA gene pyrosequencing. It was observed that abundance of *Bacteroidetes* decreased in constipated patients (mostly *Prevotella* species) and presence of several families and genera of the phylum *Firmicutes* increased [29]. Differences between our findings and those from the study including obese controls may be explained by this specific phenotype, as obesity is associated with a particular intestinal microbiota composition [30].

An explanation for the identified discriminative species detected in the present study might be the ISpro technique. This technique is a non-selective detection method, allowing to unravel the highly complex intestinal microbiota composition down to species level [15,31]. It has been shown that usage of different microbiota detection techniques may influence outcome, preventing from reliable comparison between data sets when different methods are used [32]. Since analysis of the control group was performed with IS-pro [16], we selected the same method to describe microbiota composition of the study group. In a recent study, we have shown that intestinal microbiota characterization by IS-pro and 454-pyrosequencing presented comparable results [16]. In contrast to earlier reports using 16 S ribosomal RNA gene sequencing [28], we could differentiate constipated subjects from healthy controls on fecal microbiota composition. Possible explanation for this finding might be, next to a different detection technique, differences in patient characteristics (particularly, 12/25 constipated subjects also suffered from IBS symptoms) [28]. In our study, the two study groups could be discriminated with 82% accuracy by using detailed supervised learning methods, while acceptable classification could not be reached by unsupervised clustering methods and PCoA. Supervised machine learning is in particular useful for pattern recognition in highly complex data sets, like intestinal microbiota studies. Supervised classification models are applied to obtain information from training data which can subsequently be used to direct the correct category labels to novel inputs (in this case new fecal samples). Furthermore, this model can be used to assess which species are most discriminative between subgroups. Supervised classifiers have recently been shown effective for microbiota classification, both for the selection of highly discriminative species, and for development of models that enable accurate classification of unlabeled data [33]. For example, comparative observations to our findings have recently been done in a study comparing microbial signatures of pediatric IBD patients and controls [34]. Using supervised statistical models, major differences between patients and controls were captured, dramatically increasing diagnostic accuracy, while unsupervised models did not allow for classification. The authors underlined the particular utility of such models in small-to-medium datasets with high variability. Possible explanation for the lack of discrimination between constipation and controls using standard statistical methods, is that constipation is only weakly associated with particular species or intestinal microbial composition, which even may be masked by the commonly high inter-individual variability. Additionally, constipation affects a clinically heterogeneous population, precluding straightforward nosologic classification. It may therefore be hypothesized that certain subgroups of constipated children have dissimilar microbial signatures. In these series, no correlation has been observed considering microbial composition and specific clinical characteristics, such as withholding behavior, fecal incontinence, or frequency of bowel movements.

Linking altered microbial signatures to specific subgroups, if present, may be helpful to select subjects who potentially benefit from microbiota-based interventions such as prebiotics or probiotics. Whether microbiota-based interventions in defined subgroups should be aimed at recovery of microbial imbalance, and if so, how this could be attained, remains unclear. The revised version of Koch’s postulates by Fredricks and Relman include the statement that ‘the nature of the microorganism inferred from the available sequence should be consistent with the known biological characteristics of that group of organisms’[35]. Obviously, the exact function of the discriminative species in the present study is mostly unknown and targeting at restoration of microbial disruption at the level of individual species would probably be a too simplistic approach.

Strengths of the current study are the relatively large number of included subjects, discontinuation of laxatives prior to sample collection and the use of a microbiome-wide DNA-based profiling method. Our study also had limitations. The present study was performed in two referral centers for children with functional constipation, which may induce inclusion bias. However, children with more profound disturbance of colonic motility, reflected by strongly reduced bowel movements frequency, had comparable microbiota patterns as affected subjects with less disturbed motility. Another limitation is that we performed microbiota analysis on fecal samples, which differs substantially from the mucosa-associated microbial fingerprint [36]. Since endoscopy is not routinely performed in constipated children, large scale harvesting of mucosal samples is not easily employed in this particular population. Furthermore, dietary intake was not assessed in the study groups, increasing the likelihood of a type I error, although geographically the population was derived from a relatively restricted area in which a more or less common culture and diet was to be anticipated.

The cross-sectional design of this study precluded to assess whether observed microbial differences were causative or merely consequence of constipated state. Future studies are needed to externally validate our findings, focusing on subgroups within the heterogeneous population of constipated children, and preferably using a longitudinal design, to analyze the relationship between intestinal microbial misbalance and clinical symptoms.

In conclusion, we observed that none of the standard unsupervised statistical methods allowed for microbiota-based discrimination of children with functional constipation and healthy controls. After applying a supervised mathematical learning model, both groups could be discriminated with 82% accuracy. Optimization of therapeutic microbiota-based interventions in constipated children warrants further characterization of microbial signatures linked to clinical subgroups of functional constipation.
